# Supplementary material for: Topological structures and syntenic conservation in sea anemone genomes
Source: Nat Commun. 2023 Dec 13;14:8270. doi: 10.1038/s41467-023-44080-7 (PMC10719294; doi:10.1038/s41467-023-44080-7)
Supplement: Supplementary file 3 — Description of Additional Supplementary Files [file 41467_2023_44080_MOESM3_ESM.pdf]

Description of Additional Supplementary Files

Supplementary Data 1
Description: Summary of repetitive sequence content in the N. vectensis and S. callimorphus genomes reported by RepeatMasker.

Supplementary Data 2
Description: Chromosome-wise abundance and enrichment of repeat classes in N. vectensis.

Supplementary Data 3
Description: Chromosome-wise abundance and enrichment of repeat families in N. vectensis.

Supplementary Data 4
Description: Chromosome-wise abundance and enrichment of repeat classes in S. callimorphus.

Supplementary Data 5
Description: Chromosome-wise abundance and enrichment of repeat families in S. callimorphus.

Supplementary Data 6
Description: Genomic locations of a subset of the ANTP, PRD, SINE, TALE and HNF class homeobox genes in the N. vectensis genome.

Supplementary Data 7
Description: Table of correspondence between N. vectensis cloned complete CDS sequences in the NCBI database and NV2 models.

Supplementary Data 8
Description: Ultraconserved Noncoding Elements in the N. vectensis genome.

Supplementary Data 9
Description: Genes contained in the cnidarian ancestral linkage groups.

Supplementary Data 10
Description: Genes contained in the bilaterian ancestral linkage groups.

Supplementary Data 11
Description: Genes contained in the metazoan ancestral linkage groups.

Supplementary Data 12
Description: Number of microsyntenic blocks in deuterostomes, spiralians and cnidarians.

Supplementary Data 13
Description: Relationship between the ALGs predicted in this work and those in Simakov et al 2022. Pairs of metazoan ALGs are included that share at least 5 genes between the two studies in all used genomes, based in Data File S1 in that work [10.1126/sciadv.abi5884].

Supplementary Data 14
Description: Data sources for ATAC-seq.

Supplementary Data 15
Description: Data files required to reproduce the analyses. See github repository https://github.com/nijibabulu/cnidariangenomes for details.
