# Supplementary material for: Topological structures and syntenic conservation in sea anemone genomes
Source: Nat Commun. 2023 Dec 13;14:8270. doi: 10.1038/s41467-023-44080-7 (PMC10719294; doi:10.1038/s41467-023-44080-7)
Supplement: Supplementary file 5 — Reporting Summary [file 41467_2023_44080_MOESM5_ESM.pdf]

nature portfolio

Corresponding author(s): Ulrich Technau and Matthew C. Gibson

Last updated by author(s): Aug 14, 2023

Reporting Summary

Nature Portfolio wishes to improve the reproducibility of the work that we publish. This form provides structure for consistency and transparency in reporting. For further information on Nature Portfolio policies, see our Editorial Policies and the Editorial Policy Checklist.

Statistics

For all statistical analyses, confirm that the following items are present in the figure legend, table legend, main text, or Methods section.

n/a	Confirmed	
☐	☒	The exact sample size (*n*) for each experimental group/condition, given as a discrete number and unit of measurement
☒	☐	A statement on whether measurements were taken from distinct samples or whether the same sample was measured repeatedly
☐	☒	The statistical test(s) used AND whether they are one- or two-sided<br>*Only common tests should be described solely by name; describe more complex techniques in the Methods section.*
☒	☐	A description of all covariates tested
☒	☐	A description of any assumptions or corrections, such as tests of normality and adjustment for multiple comparisons
☐	☒	A full description of the statistical parameters including central tendency (e.g. means) or other basic estimates (e.g. regression coefficient) AND variation (e.g. standard deviation) or associated estimates of uncertainty (e.g. confidence intervals)
☐	☒	For null hypothesis testing, the test statistic (e.g. *F*, *t*, *r*) with confidence intervals, effect sizes, degrees of freedom and *P* value noted<br>*Give P values as exact values whenever suitable.*
☒	☐	For Bayesian analysis, information on the choice of priors and Markov chain Monte Carlo settings
☒	☐	For hierarchical and complex designs, identification of the appropriate level for tests and full reporting of outcomes
☒	☐	Estimates of effect sizes (e.g. Cohen's *d*, Pearson's *r*), indicating how they were calculated

Our web collection on statistics for biologists contains articles on many of the points above.

Software and code

Policy information about availability of computer code

Data collection	Data were downloaded using the sratoolkit version 2.11.0 where possible, otherwise from links supplied by databases and authors.
Data analysis	Software and versions used to analyze the data are enumerated detail in in the Materials and Methods section. Custom code used to analyze the data are available at https://github.com/nijibabulu/cnidariangenomes

For manuscripts utilizing custom algorithms or software that are central to the research but not yet described in published literature, software must be made available to editors and reviewers. We strongly encourage code deposition in a community repository (e.g. GitHub). See the Nature Portfolio guidelines for submitting code & software for further information.

Data

Policy information about availability of data

All manuscripts must include a data availability statement. This statement should provide the following information, where applicable:

- Accession codes, unique identifiers, or web links for publicly available datasets
- A description of any restrictions on data availability
- For clinical datasets or third party data, please ensure that the statement adheres to our policy

All raw data and the assembled genomes are available via the National Center for Biotechnology Information under the accession PRJNA667495 at the following URL: [https://www.ncbi.nlm.nih.gov/bioproject/PRJNA667495]

Field-specific reporting

Please select the one below that is the best fit for your research. If you are not sure, read the appropriate sections before making your selection.

☒ Life sciences ☐ Behavioural & social sciences ☐ Ecological, evolutionary & environmental sciences

For a reference copy of the document with all sections, see nature.com/documents/nr-reporting-summary-flat.pdf

Life sciences study design

All studies must disclose on these points even when the disclosure is negative.

Sample size	Sample sizes for all genomes were 1, except where validating sequencing was performed. In this case sample sizes were 2 but no statistical tests were performed.
Data exclusions	We did not exclude any data that was available
Replication	For all species only one genome was available therefore no replication was possible. For validating nematostella data no statistical tests were performed, however 2 successful replicates of independent validation were performed for sequencing analysis.
Randomization	No randomization was necessary
Blinding	No blinding was necessary.

Reporting for specific materials, systems and methods

We require information from authors about some types of materials, experimental systems and methods used in many studies. Here, indicate whether each material, system or method listed is relevant to your study. If you are not sure if a list item applies to your research, read the appropriate section before selecting a response.

Materials & experimental systems

n/a	Involved in the study
☒	☐ Antibodies
☒	☐ Eukaryotic cell lines
☒	☐ Palaeontology and archaeology
☐	☒ Animals and other organisms
☒	☐ Human research participants
☒	☐ Clinical data
☒	☐ Dual use research of concern

Methods

n/a	Involved in the study
☒	☐ ChIP-seq
☒	☐ Flow cytometry
☒	☐ MRI-based neuroimaging

Animals and other organisms

Policy information about studies involving animals; ARRIVE guidelines recommended for reporting animal research

Laboratory animals	Nematostella vectensis laboratory strain (F1 CH2xCH6) was used.
Wild animals	Scolanthus callimorphus was collected at Île Callot, Carantec, France. Age and sex are unknown. They were transported in a 50ml centrifuge tube containing salt water at room temperature to Vienna where they were kept in a sand aquarium in a 20 degrees celsius room prior to sacrificing for genomic DNA.
Field-collected samples	*For laboratory work with field-collected samples, describe all relevant parameters such as housing, maintenance, temperature, photoperiod and end-of-experiment protocol OR state that the study did not involve samples collected from the field.*
Ethics oversight	No ethics oversight was necessary. No permits were necessary to collect the samples

Note that full information on the approval of the study protocol must also be provided in the manuscript.
